# The impact of self-employment on mental health of the younger elderly in China

**DOI:** 10.1186/s12877-023-03948-5

**Published:** 2023-05-08

**Authors:** Deshui Zhou, Qianqian Zhan, Lele Li

**Affiliations:** 1 School of Finance and Public Management, Anhui University of Finance & Economics, Bengbu, Anhui Province China; 2 School of Labor and Human Resources, Renmin University of China, Beijing, China

**Keywords:** Younger elderly, Self-employment, Mental health, Self-realization

## Abstract

**Background:**

With the prolongation of the life expectancy of the Chinese population and the intensification of the aging process of the population, the mental health problems of the elderly have become increasingly prominent. This study aims to explore whether self-employment can promote and how to promote the mental health of the elderly.

**Method:**

Based on the 2018 China Longitudinal Aging Social Survey (CLASS) data, this paper uses OLS model and KHB method to verify the impact of self-employment on the mental health of the younger elderly and its mechanism.

**Results:**

The results indicate that self-employment can significantly reduce the depression tendency of the younger elderly and promote their mental health. Heterogeneity analysis shows that self-employment has a more significant positive impact on the mental health of the younger elderly who are self-rated healthy, free of chronic diseases and low-level medical service utilization. The mechanism shows that self-employment can indirectly improve the mental health of the younger elderly through income growth effect and self-worth realization effect, in which the self-worth realization effect is greater than the economic effect. It illustrates that with the development of China’s economy, the elderly are pursuing more intrinsic values brought by self-employment than economic benefits.

**Conclusion:**

In view of the above research results, it is suggested to encourage the elderly to actively participate in social activities, provide policy support for the younger elderly to engage in self-employment, increase government support as well as health guarantee level, and improve the subjective initiative of the elderly to participate in self-employment, so that the society can truly realize the healthy aging of “being useful and productive for the elderly”.

## Introduction

Since the 18th National Congress of the Communist Party of China, remarkable achievements have been made in China’s economic and social development, people’s living standards have gradually improved, and national life expectancy has also been continuously extended. By 2020, China’s average life expectancy has reached 77.93 years, and it is expected to continue to increase by 1 year by 2025. However, the improvement of life expectancy is accompanied by the deepening of population aging. In order to actively respond to the aging population, it is necessary to practice a positive outlook on aging, encourage the elderly to continue to play a role, and support the elderly to engage in business and production activities in accordance with the law and regulations. Self-employment, as an important form of individual operation, has become a common choice for workers in the era of digital economy [1]. Self-employment is a broadly defined concept. Steinmetz and Wright [2] defined self-employment as “a job that obtains part or all of its income through its own labor rather than selling its own labor to the employer to obtain wages”. The Organization for Economic Cooperation and Development (OECD) classifies self-employed persons into three categories: self-employed persons without employees, employers with employees, and contributing family members without fixed remuneration [3]. In short, self-employment means that workers employ themselves to engage in non-agricultural profit-making work, regardless of whether they employ others. For the elderly, they gradually quit their jobs because of their age [4], while self-employment offers them a feasible path to return to the labor market. According to the “Pensions at a Glance 2019: OECD and G20 Indicators” released by the OECD in 2020, the proportion of self-employed elderly people to all working elderly people is 38%. In addition, according to the itemized data of the China Census Yearbook-2020 survey, among the employed persons in various industries, the self-employed industries such as agriculture, forestry, animal husbandry and fish account for the highest proportion of the elderly employment in China, accounting for 66.16% of the elderly employment population. It can be seen that self-employment is indeed becoming increasingly common among the elderly.

However, the mentality of the elderly will also change with the increase of age and the transformation of lifestyle, and negative emotions such as depression and anxiety become increasingly common [5]. China National Mental Health Development Report (2019–2020) pointed out that nearly one-third of the elderly are depressed, and the mental health problems of the elderly are becoming increasingly prominent, which has become a topic worthy of deep concern. As workers reach the retirement age, they can relieve their labor obligations and quit their jobs [6],and the status of the elderly is changed from busy workers to leisure retirees [7]. Self-employment enables the status of the elderly workers to continue and continue to play their own value [8], providing new ideas for improving the mental state of the elderly. Therefore, taking self-employment as the breakthrough point, studying the mental health of the elderly is of great significance to promote the elderly to establish a positive attitude towards aging and realize the healthy aging in China.

So, will self-employment affect the mental health of the elderly? If this influence exists, what is its mechanism? Based on the 2018 China Social Tracking Survey of the Elderly (CLASS) data, this paper uses OLS model and KHB method to verify the impact of self-employment on the mental health of the younger elderly and its mechanism. The marginal contributions of this paper are as follows: First, focusing on the younger elderly, and using the data of the 2018 China Social Tracking Survey of the Elderly to empirically test the impact of self-employment on the mental health of the younger elderly, so as to provide new ideas for improving the mental health level of the elderly and bolstering the active aging; Secondly, from the theoretical aspect, this paper puts forward the channels of self-employment affecting the mental health of the younger elderly, uses microscopic data to construct intermediary variables for testing, and compares the contributions of different channels, revealing the internal needs of the younger elderly in more details.

## Literature review

The mental health of the elderly is an important part of the welfare of the elderly. For a long time, the academic circles have kept close attention and strong interest in the mental health of the elderly, and explored the influence of various factors on the mental health of the elderly from the perspectives of family, society and economy. At the family level, inter-generational support can not only improve the mental health of the elderly from their children’s care [9], but also promote the mental health of the elderly through “reverse feeding” [10]. At the social level, the more social capital, social support and social participation the elderly individuals have, the lower the degree of depression of the elderly will be [11, 12], and social isolation will have a negative impact on the mental health of the elderly by causing anxiety and depression [13]. In addition, the economic situation is also an important influencing factor that can’t be ignored. Poverty will cause mental health problems such as cognitive degradation and depression of the elderly, and the mental state will be significantly improved after income growth [14].

With the increase of life expectancy, the elderly still remain active in society, and continue to play their own value to create wealth for the society [15]. Employment is one of the important activities of social participation [16, 17], and studies have shown that employment is an important factor affecting the mental health of older adults. There is no unanimous conclusion on the impact of employment on the mental health of the elderly in the existing research, which can be roughly divided into two kinds of views: one kind of view holds that employment promotes the mental health of the elderly. Compared with the elderly who do not participate in labor, the elderly who engage in labor have a lower degree of depression [5]. Moreover, the loneliness of retirees is significantly higher than that of participants who are still working [18], and even the elderly who re-enter the job market after retirement, their depression tendency is significantly reduced [19, 20]. The other view is that employment reduces the mental health of the elderly. Maintaining employment after retirement has a significant negative impact on the life satisfaction and happiness perception of the elderly, which is more prominent among female elderly people [21].

Meanwhile, some scholars have made a more detailed division of employment types, and discussed the influence of self-employment types on the mental health of the elderly. Numerous studies have proved that self-employed people are healthier, happier and more satisfied at work than employed people [22, 23], because they have a high degree of autonomy and flexibility in their work, which can effectively relieve stress and reduce negative emotions [24]. However, contrast exists among self-employed groups, and is indicated in the phenomenon that self-employed people with employees have higher life satisfaction than those without employees [25]. There are also scholars who have verified it from the verso angle. Patel et al. [26] used the data from the European Survey of Health, Aging and Retirement (SHARE) to examine the relationship between self-employed elderly workers and depression symptoms, and found that self-employment is negatively correlated with depression of elderly workers, but the benefits of self-employment to the mental health of the elderly is probably limited. People who still engage in self-employment beyond the standard retirement age may experience a higher degree of depression. However, some studies have come to the opposite conclusion that self-employment has negative effects on the general health and mental health of the elderly, while employment has positive effects on the physical and mental health of the elderly [27]. On the whole, most scholars believe that self-employment can keep the elderly active, boost their happiness and improve their mental health.

So through which channels does self-employment affect the mental health of the elderly? Self-employment can bring economic and non-economic benefits, which is an important path of the relationship between self-employment and mental health [28]. Economic welfare is manifested through the fact that self-employment can bring the increase in income, the decline in economic burden and the alleviation in anxiety, depression and mental disorders [29]; Non-economic welfare is reflected in the fact that self-employed people have more autonomy and flexibility in their work, helping them overcome or avoid working pressure. This control over work plays an intermediary role in the relationship between self-employment and mental health [30]. For the elderly, not only will self-employment will promote their mental health economically, but also respect for non-economic benefits such as needs, social participation, life satisfaction and happiness are the transmission path that affects self-employment on the mental health of the elderly [20][26].

To sum up, at present, the academic circles have conducted in-depth and detailed research on the mental health of the elderly from various perspectives, and the mental health effects caused by employment have arisen the scholars’ attention to a certain extent, providing relevant reference for this study, but room for expansion still exists. First, little attention has been paid to the impact of self-employment on the mental health of the younger elderly, especially since China has entered a stage of deep aging, the study on the effect of re-employment of the younger elderly deserves attention and discussion. Second, having empirically examined the impact of the employment of the elderly on their mental health, the existing research seldom cogitates on the general age restriction of the employment of the elderly, which can’t well explain practicalities and implement policies. Third, concerning the operation mechanism, previous studies lack analyses for the important pathway to the realization of self-worth, with deficiency of relevant theoretical support and empirical test. This paper aims to enrich the research space of self-employment and mental health of the elderly.

## Theoretical analysis and research hypothesis

According to the Activity Theory, the elderly population can engage in social work, and the higher the activity level, the easier it is for the elderly to feel satisfied with life and adapt to the society, thus reducing the degree of depression [31]. For the young people who have just stepped into the old age, they still have the ability to work after retirement [32], they can continue to participate in social activities and sustain a lifestyle with a high activity level, so as to maintain their mental health. Although the employment of the elderly can bring many benefits in theory, based on practical considerations, on the one hand, because of rational economic choices, employers in the labor market are often reluctant to hire the elderly who has low production efficiency [15], so the elderly are unlikely to re-work through employment, and thus self-employment becomes a realistic way for them to participate in employment. On the other hand, self-employment enjoys the advantage of high flexibility. Workers can arrange their working hours and contents independently according to their own conditions [30]. Compared with employed employment, self-employment requires lower physical energy consumption for the elderly [26] Therefore, self-employment may have a positive impact on the mental health of the younger elderly.

With the gradual improvement of China’s social elderly care security system, although people’s concept of raising children to provide against old age has not fundamentally changed, the people’s general concept of old-age care has also undergone significant changes, and the concept of “independent old-age care” has gradually taken shape [33]. Moreover, the nation continuously reinforcing the individual and independent responsibility of elderly care, the economic dependence of the elderly on their children has gradually decreased. At the same time, due to the economic slowdown and the aggravation of aging, the elderly care security system is also facing great pressure. The economic support to the elderly brought by endowment insurance is feeble, and the elderly need to gain economic income through self-reliance. A large number of studies have proved that self-employment can augment the income level of workers [34, 35], and the improvement of economic status can reduce the depression probability of the elderly, relieve anxiety and promote mental health [20]. Therefore, self-employment may possess an influence mechanism of increasing income to improve the mental health of the younger elderly.

An important reason for depression among the elderly is the change of identity and role [36, 37]. According to the Role Theory in Social Psychology, everyone in society plays a corresponding role in the particular social position. Society and others have requirements for individuals corresponding to their social position, namely social expectations [38]; Everyone has to fulfill the obligations entrusted by society, exercise power and assume role responsibility. Such social role behavior satisfies people’s highest demand for the realization of self-worth. Labor participation is a signal way for people to realize their self-worth [39]. However, in the old age, people gradually withdraw from the labor market, and their core social role changes from the “source” of creating value to the “burden” of consuming value. This change in role has an infinite impact on the psychology of the elderly, inclining the elderly to engender depression, loneliness and anxiety. It is often difficult for the elderly to adapt to such change in roles, not only because they have lost the social status and power of their original roles, but also because they have lost their feelings of self-affirmation.

The elderly in the leisure state express an intense demand for spiritual satisfaction [40]. Although the deterioration of physical function is an unavoidable process, they are still reluctant to leave the society and become a burden on the society, hoping to be accepted by the society and continuously realize their self-worth instead. And this kind of spiritual appeal can’t be realized completely by others. Therefore, the younger elderly who have just stepped into retirement need to participate in the society [41], to play a new role to return to the dominant position in society from the social object position, to constantly make contributions to the society, and to meet the needs of self-realization, in order to have their own values improved [42]. For self-employed employment can provide challenges, socializing and feelings of being valued or needed [43], self-employed people often experience a conscious and fervent positive-feeling of pursuing their goals [44]. Self-employment of the elderly enables the role of workers to remain in old age [18], conducive for the elderly to regain, maintain and even strengthen their sense of self-worth, realize their self-worth and bring psychological satisfaction. Therefore, self-employment may improve the mental health of the younger elderly by realizing their self-worth.

To sum up, this paper puts forward the following research hypotheses:

### Hypothesis 1

Self-employment has a positive impact on the mental health of the younger elderly.

### Hypothesis 2

Self-employment may have an impact on the mental health of the younger elderly by increasing their income and self-worth.

## Method

### Data sources

This paper uses the data of the 2018 China Longitudinal Aging Social Survey(CLASS). The China Social Tracking Survey of the Elderly is a national social survey project jointly designed and implemented by the Population and Development Research Center of Renmin University of China and the Institute of Gerontology, the third round of which was in 2018. The 2018-survey collected data on the backgrounds in individuals, families, society and economy of the elderly in China, which can well reflect the basic situation of the elderly in China and the problems and challenges they are facing in the aging process. This paper studies the self-employment of the elderly. Considering the health human capital situation of the elderly, the elderly of advanced age are no longer suitable for re-employment. Therefore, this paper selects the data of the younger elderly aged 60–69 as the samples for empirical analysis, in order to ensure the integrity of the variable data used in the article, we have eliminated the invalid samples whose answers to the variable questions involved in the article are “don’t know”, “refuse to answer” and “can’t answer”, and finally get the effective sample size of 3914. In addition, after screening the samples, although the data volume of this article has decreased, the sample coverage still covers the vast majority of provinces in the country, with strong representativeness.

### Selection of variables

#### Explained variable

The explained variable of this paper is the mental health of the elderly, which is measured by the Self-rating Depression Scale (CES-D) of the Center for Epidemic Investigation. In this study, the simple edition of the Self-rating Depression Scale (CES-D9) in the CLASS questionnaire was used to measure depression tendency. CESD-9 consists of nine questions about mental health, including three questions on positive emotions and six on the negative emotions. The three questions are as follows: in the past week, “Have you been in a good mood?“ “Have you had a good life?“ “Have your life been full of interest?“. The six questions are as follows: in the past week, “Have you ever felt lonely?“ “Have you been sad?“ “Have you been reluctant to eat?“ “Have you had trouble in sleeping?“ “Have you ever felt useless?“ “Have you been isolated by others?“. The answers of each question reflecting the degree of depression were assigned to 0, 1 and 2 from low to high, and the total score was calculated by adding up the scores of 9 questions, and was used to describe the mental health status of the elderly sample individuals. The values ranged from 0 to 18, and the higher the score, the worse the mental health status is of the elderly. The internal consistency coefficient of the CES-D9 scale in this paper is 0.69. In the exploratory study, the internal consistency coefficient can be less than 0.7, but should be greater than 0.6. The internal consistency coefficient of the CES-D9 scale in this paper is greater than 0.6 and close to 0.7, which to some extent shows that the scale has good reliability and the research in this paper is reliable.

#### Explanatory variables

The core explanatory variable of this paper is self-employment of the elderly. Self-employment is the opposite form of employment, which means that workers employ themselves to engage in non-agricultural and profitable work, including self-employed, non-unit freelancers and some workers who do not work for themselves. According to the question: “What do you mainly do at present?“, the study defines the answer “self-employed, freelancer, or private business owner” as self-employed employment group, and assign it to 1, otherwise, assign it to 0. As can be seen from Table 1, the proportion of the young elderly who participate in self-employment is only 4.1% of the total sample, indicating that the overall number of the young elderly who re-employed through self-employment is low in the interviewed sample.

It should be pointed out that self-employment can be divided into two categories: survival self-employment and opportunity self-employment [45], and can be divided into different types, such as operating stores, grocery shopping and gardening. The existing research can also provide some reference for this purpose. For example, Zhao and Zhou [45] pointed out that both the opportunistic self-employed and the survival self-employed have significant health promotion effects, but the self-rated health effect of the opportunistic self-employed is higher than that of the survival self-employed. Zhou and Wen [23] also pointed out that both opportunity self-employment and survival self-employment can significantly reduce the degree of health inequality, and the impact of opportunity self-employment is higher than that of survival self-employment. However, the type of self-employment is not distinguished in the Chinese Social Tracking Survey of the Elderly (CLASS) used in this article, so this article will do further research in the future.

#### Control variables

Control variables are mainly divided into three categories: First, individual characteristic variables, including gender, marriage, party membership, household register, education level and chronic diseases. Among them, the marital status is divided into two categories and assigned: “married with spouse” =1, “widowed, divorced or unmarried” =0; Being a member of the Communist Party of China is assigned to 1, no to 0; Assign the household register variables as “non-agricultural registered permanent residence, change from non-agricultural registered permanent residence to unified household registration” =1, “agricultural registered permanent residence, change from agricultural registered permanent residence to unified household registration” =0; Education level is classified into five variables: “primary school and below = 1, junior high school = 2, senior high school and technical secondary school = 3, junior college = 4, undergraduate and above = 5”; Chronic disease = 1, no chronic disease = 0. Next are variables of family economic characteristics, including family size, financial support from children, emotional comfort from children and personal income. Family size refers to the number of people living together, including respondents; according to the questionnaire: “In the past 12 months, have your children given you (or your living spouse) money, food or gifts? How much are these things worth?“, the variable of financial support is set as a binary variable, and assign the answer “never given” to 0, and the rest to 1; according to the questionnaire, “Do you think your children don’t care enough about you?“, emotional comfort variable assigns the answer “never” to 1, and the answer “occasionally, sometimes, often” to 0; and take the natural logarithm of the total personal income in the past 12 months. The third is social characteristic variables, including endowment insurance, social network, activity facilities and Internet use. The variable of endowment insurance will enjoy the basic pension insurance of enterprise employees, institutions and institutions and urban and rural residents, and any one of them will be assigned to 1, and none of them will be assigned to 0; The society is measured by the number of friends who can help it when needed; Community activities and facilities are assigned a value of 1, and no value of 0; Internet usage can be divided into two categories: “surfing every day, at least once a week, at least once a month, and several times a year” =1, and “never surfing the Internet” =0. The definitions and descriptive statistics of each variable are shown in Table 1.

**Table 1:** Descriptive statistics of main variables in this paper

Variable	Variable definition	Observed value	Average value	Standard deviation
CESD score	The total score of 9 questions, ranging from 0 to 18.	3914	6.088	3.098
Self-employment	Yes = 1, no = 0	3914	0.041	0.199
Gender	Male = 1, female = 0	3914	0.520	0.5
Marriage	Married spouse = 1, widowed, divorced, unmarried = 0	3914	0.813	0.39
Party member	Whether it is party member of the Communist Party of China, yes = 1, no = 0	3914	0.035	0.184
Household register	Agricultural registered permanent residence, from non-agricultural registered permanent residence to unified resident account = 1, agricultural registered permanent residence, from agricultural registered permanent residence to unified resident account = 0.	3914	0.48	0.5
Level of education	Primary school and below = 1, junior high school = 2, senior high school, technical secondary school = 3, junior college = 4, undergraduate and above = 5.	3914	1.634	0.803
Chronic ailment	Yes = 1, no = 0	3914	0.727	0.445
Family size	Number of families living together, including myself	3914	2.638	1.214
Financial support	Whether children provide financial support to parents, yes = 1, no = 0	3819	0.889	0.314
Emotional comfort	Whether children care about their parents, yes = 1, no = 0	3819	0.811	0.391
Income	The total income of individuals in the past 12 months is logarithmic.	3862	8.247	1.318
Endowment insurance	Whether to enjoy basic old-age insurance, yes = 1, no = 0	3914	0.804	0.397
Social network	The number of friends who can provide assistance when needed, none = 0, one = 1, two = 2, three to four = 4, five to eight = 4, nine and above = 5	3914	2.069	1.225
activity facilities	Whether there are activities or facilities in the community, yes = 1, no = 0.	3914	0.739	0.439
Use the internet	Use the internet, yes = 1, no = 0.	3914	0.332	0.471

Based on the selected sample data, this article reports the CESD scores of the elderly at different values of each variable. As shown in Table 2, the average CESD of young and elderly people who participate in self-employment is 5.333, while the average CESD of young and elderly people who do not participate in self-employment is 6.121. The mental health status of self-employed elderly people is better than that of non self-employed elderly people. In addition, the mental health of young and elderly people who are married, have a spouse, have a non agricultural household registration, have a high level of education, provide emotional comfort to their children, have a high income, have a large social network, have activity facilities in the community, and use the Internet are also significantly better.

**Table 2:** Comparison of the mean CESD between self-employed and non self-employed young and elderly people

Variable		CESD mean	CESD standard deviation
Self employment	Self employment	5.333	3.232
	Not Self Employed	6.121	3.088

Note: Due to space limitations, CESD mean comparisons for other variables will not be listed here

### Model setting

#### Benchmark regression: ordinary least squares (OLS) model

As the dependent variable of mental health is a continuous variable in this paper, OLS model is adopted for basic regression. The benchmark regression model is as follows:

1 $${MH}_{i}={\alpha }_{0}+{\beta }_{1}{SE}_{i}+{\beta }_{2}{X}_{i}+{\mu }_{i}$$

Among them, (Mental Health) is the explained variable of mental health in this paper, represents self-employment which is the core explanatory variable of this paper,${\beta }_{1}$ is the corresponding coefficient, and is also the main coefficient of this paper.${X}_{i}$ manifests the control variables of this study, including individual characteristics, family characteristics and employment characteristics, ${\beta }_{2}$ is the coefficient to be estimated of the control variable; ${\alpha }_{0}$ is a constant term; ${\mu }_{i}$ is a random error term.

#### Robustness test: propensity score matching

In reality, the self-employment of the younger-elderly may not happen randomly, and the self-employment behavior is likely to be influenced by the characteristics of the elderly themselves; in other words, a certain self-selection problem exists in whether the younger elderly are self-employed. Therefore, in order to control the endogenous problems caused by this self-selection bias, this paper further uses the Propensity Score Matching method to estimate the net effect of self-employment on the mental health of the younger elderly. The “counterfactual” framework constructed by Propensity Score Matching is used to estimate the mental health status of the younger self-employed elderly when they are not self-employed, and then compared with their mental health status when they are self-employed, so as to obtain the Average Treatment Effect for the Treated Group (ATT). The specific model is as follows:

2 $$ATT=E\left({Y}_{i1}|{D}_{i}=1\right)-E\left({Y}_{i0}|{D}_{i}=1\right)$$

In formula (2), ${Y}_{i1}$ indicates the mental health status of the i-th younger elderly when they choose self-employment, and ${Y}_{i0}$ indictaes the mental health status of the i-th younger elderly who does not choose self-employment on assumptions. For it is hard to know the mental health value of the young elderly who originally chose self-employment when they didn’t, the numerical feature is estimated by “counterfactual” $E\left({Y}_{i0}|{D}_{i}=1\right)$. ATT in formula (2) expresses the average processing utility, which implies the net effect of self-employment on the mental health of the younger elderly.

#### Intermediary effect test: KHB model

More flexible in model setting, the KHB decomposition method proposed by Karlson et al.(2012), is adopted to examine the mechanism of self-employment affecting the mental health of the younger elderly. This method decomposes the total effects of variables into direct effects and indirect effects. The predicted model is as follows:

3 $${MH}_{i}={\alpha }_{F}+{\beta }_{F}{SE}_{i}+{\gamma }_{F}{Med}_{i}+{\delta }_{F}{X}_{i}+{\omega }_{i}$$

Among them, ${Med}_{i}$ expresses the intermediary variable represented in this article, ${\beta }_{F}$ is the independent variable coefficient, ${\gamma }_{F}$ is the intermediary variable coefficient, ${\delta }_{F}$ is the control variable coefficient, and ${ \omega }_{i}$ is the random error term. The mental health status of the younger elderly can be indirectly influenced by self-employment through intermediary variables. At this time, the direct effect of self-employment on their mental health is expressed as follows:

4 $${b}_{F}=\frac{{\beta }_{F}}{{\sigma }_{F}}$$

The total effect of self-employment on mental health is expressed as:

5 $${b}_{R}=\frac{{\beta }_{R}}{{\sigma }_{R}}$$

The indirect effect of self-employment on mental health through intermediary variables are:

6 $${b}_{R}-{b}_{F}=\frac{{\beta }_{R}}{{\sigma }_{R}}-\frac{{\beta }_{F}}{{\sigma }_{F}}$$

## Results

### Analysis of the results of benchmark regression model

The software used for data processing in this paper and its version are StataSE 17. This paper analyzes the influence of self-employment on the mental health of the younger elderly by gradually adding control variables. Table 3 reports the benchmark regression results based on OLS model. In model 1, the control variables of individual characteristics are added, and the results show that self-employment reduces the depression tendency of the younger elderly at the statistic level of 1%. In model 2, the control variable of family economic characteristics is further added into the model, and the result is also significantly negative at the statistic level of 1%. In model 3, after adding social characteristics control variables into the model, the result remains stable, which implies that self-employment has an inhibitory effect on the depression tendency of the younger elderly. The regression results show that self-employment promotes the mental health of the younger elderly, and thus hypothesis 1 is verified.

In the economic sense, it can be yielded from the results of Model 3 that after controlling all variables, choosing to participate in self-employment will significantly reduce the depression level of the younger elderly by 1.48 units. And namely, self-employment will improve the mental health of the younger elderly by 1.48 units. The CESD score of the younger elderly in the sample is 6.088, which means that participating in self-employment can reduce the depression score of the younger elderly by around 24%. From the perspective of active aging, the involvement in self-employment of the younger elderly has helped to promote mental health of the younger elderly and facilitate the active aging of the society. The economic effect is remarkable.

**Table 3:** Measurement and estimation results of self-employment on mental health of young elderly people

Variable	Model 1	Model 2	Model 3
Self-employment	-0.689***	-1.634***	-1.480***
	(0.262)	(0.268)	(0.274)
Gender	-0.006	-0.014	-0.060
	(0.096)	(0.096)	(0.093)
Marriage	-0.722***	-0.710***	-0.621***
	(0.122)	(0.123)	(0.119)
Party member	0.295	0.370	0.327
	(0.263)	(0.270)	(0.262)
Household register	0.012	0.043	0.333***
	(0.104)	(0.115)	(0.116)
Level of education	-0.556***	-0.496***	-0.268***
	(0.069)	(0.070)	(0.070)
Chronic ailment	0.475***	0.476***	0.490***
	(0.104)	(0.105)	(0.102)
Family size		-0.041	-0.047
		(0.039)	(0.039)
Financial support		-0.399***	-0.283*
		(0.150)	(0.146)
Emotional comfort		-0.591***	-0.595***
		(0.121)	(0.121)
Income		-0.157***	-0.122***
		(0.042)	(0.041)
Endowment insurance			0.304**
			(0.121)
Social network			-0.232***
			(0.039)
Activity facilities			-0.635***
			(0.112)
Use the internet			-1.271***
			(0.119)
Constant term	6.307***	8.446***	8.877***
	(0.185)	(0.415)	(0.415)
Virtual variables of provinces	control	control	control
Pseudo R2	0.13	0.153	0.201
Observed value	3914	3770	3770

Note: * * *, * * and * are significant at the statistical level of 1%, 5% and 10% respectively. The brackets are robust standard errors

In terms of individual characteristic variables, gender and party member status have no obvious influence on the mental health of the younger elderly, and marital status significantly reduces the depression tendency of the younger elderly at the statistical level of 1%. Compared with the elderly without spouses, the elderly with spouses will be relieved of their loneliness in life, ameliorating their mental health to some extent. Household registration will significantly affect the mental health of the younger elderly. The mental health of the urban registered elderly is worse than that of the rural registered elderly. The possible reason is that the urban youth in today’s society are facing onerous living pressure, and many urban younger-elderly have to provide downward intergenerational support for their children after retirement, sharing their children’s various pressures, which easily generates negative emotions such as anxiety as well as irritability, and thus reducing their mental health. The education level is significantly negative at the statistical level of 1%, which indicates that with the increase of education level, the depression tendency of the younger elderly gradually decreases. The higher the education level is, the stronger the self-adjustment ability of the elderly have, which can better dispel negative emotions and ameliorate their mental state. Chronic diseases significantly reduce the mental health level of the elderly.

In terms of family economic characteristics, family size has no significant impact on the mental health of the younger elderly, but financial support and emotional comfort from children can significantly reduce their depression. This is because children’s financial support can materially raise the living standard of the elderly, and children’s emotional support can mentally relieve the loneliness of the elderly, meet their missing emotional needs and improve their mental health. The coefficient value of personal income is significantly negative at the statistical level of 1%, indicating that the higher the income of the younger elderly in the past year has been, the lower their depression tendency will be.

In terms of social characteristics, the variable coefficient of endowment insurance is significantly positive at the statistical level of 5%, indicating that the elderly with endowment insurance are more prone to depression than those with no endowment insurance. The possible reason is that, under the influence of Chinese traditional culture, children have to wear the mantle of supporting their parents after retirement. Children’s support makes the elderly feel that their children are filial and their efforts are rewarded. However, there is a certain substitution effect between endowment insurance and children’s support. The endowment insurance weakens the degree of children’s support for their parents and reduces the positive feelings of the elderly when interacting with others. On the statistical level of 1%, the social network has reduced the depression tendency of the younger elderly. The abundant social network reflects the close relationship between the elderly and others, from which they can gain some social support, feel pleased and improve mental health. Community equipped with activity places or facilities can significantly ameliorate the mental health of the younger elderly at the statistical level of 1%. The elderly can use the places or facilities in the residential community to carry out various social activities and exercise. In these processes, the elderly not only satisfy their interests, but also expand interpersonal relationships, and also strengthen their bodies, which is conducive to maintaining positive mental health. The use of the Internet significantly promotes mental health, which is probably because the entertainment and social functions of the Internet alleviate the emptiness of the elderly, thus reducing their depression tendency.

### Robustness test

#### Change dependent variable setting

As the mental health status is measured by the total scores by nine mental health problems in the questionnaire, in order to further examine the robustness of the benchmark regression results, this paper makes a robustness test by adjusting the measurement standards of mental health status. The first method is to assign the continuous variable of the original mental health score to a binary variable. When the score is more than or equal to 6, it is defined as obvious depressive symptoms, and when the score is less than 6, it is defined as mild depressive symptoms. The second method is to divide CESD-9 into two mental health sub-indicators: the total score of three positive emotional problems and the total score of six negative emotional problems, tested from the perspective of positive and negative mental health. The re-estimation results are shown in Table 4. Column (1) uses binary variables of mental health for prediction. The predicted results show that its coefficient value is still significantly negative at the statistical level of 1%; Column (2) is the regression result of the influence of self-employment on positive emotions. The result shows that self-employment promotes the positive emotions of the younger elderly at the statistical level of 1%. Column (3) is the regression result of the influence of self-employment on negative emotions. The result shows that self-employment inhibits the negative emotions of the younger elderly at the statistical level of 1%. It can be seen that the analysis results of self-employment on mental health are consistent with the basic regression results, which indicates that the core conclusion of this paper is steady, that is, self-employment has a positive impact on the mental health of the younger elderly.

**Table 4:** Robustness test results of changing dependent variable settings

Variable	(1)	(2)	(3)
	Binary variables of mental health	Positive emotion	Negative emotion
Self employment	-0.254***	0.607***	-0.873***
	(0.044)	(0.161)	(0.211)
Constant term	0.954***	2.565***	5.442***
	(0.067)	(0.250)	(0.365)
Control variable	control	control	control
Virtual variables of provinces	control	control	control
Pseudo R2	0.159	0.152	0.095
Observed value	3770	3770	3770

Note: * * *are significant at the statistical level of 1%. The brackets are robust standard errors

#### Propensity score matching

For self-selection problems probably exist in the benchmark regression model, in this study, the Propensity Score Matching (PSM) is used for estimation to test the robustness of the results. In this study, nearest neighbor matching, radius matching and kernel matching are used to predict the samples. As is shown in Table 5, the estimated ATT value of nearest neighbor matching before matching is -1.715, and after matching, the ATT value drops to -1.516, which is significant at the statistical level of 1%. It manifests that self-employment can significantly reduce the depression tendency of the younger elderly by 1.516. In this paper, the results of radius matching and kernel matching have also been predicted. The ATT values after matching are − 1.547 and − 1.543, respectively, which are both significant at the statistical level of 1%. The conclusion is basically consistent with the nearest neighbor matching. From this point of view, the conclusion that self-employment has significantly improved the mental health of the younger elderly is steady.

**Table 5:** Self-selection Treatment of Self-employment of Young Elderly

Matching type	Matching condition	Processing group	Control group	ATT value	Standard error	T value
Nearest neighbor matching	Front matching	4.396	6.111	-1.715	0.302	-5.67***
	After matching	4.396	5.912	-1.516	0.337	-4.49***
Radius matching	Front matching	4.396	6.111	-1.715	0.302	-5.67***
	After matching	4.396	5.944	-1.547	0.299	-5.17***
Nuclear matching	Front matching	4.396	6.111	-1.715	0.302	-5.67***
	After matching	4.396	5.939	-1.543	0.299	-5.16***

Note: The nearest neighbor matching adopts 1:4 matching, the radius matching radius value is 0. 05, and the kernel matching is the default value. * * *are significant at the statistical level of 1%.

### Heterogeneity analysis

In this paper, the heterogeneous effects of self-employment on the mental health of the younger elderly are deeply investigated from three aspects: self-rated health, chronic diseases and medical service utilization. Since the research group in this paper is the elderly, whose physical function gradually degrades with the growth of age, and the level of physical health will decline to varying degrees, we want to know the difference in the impact of self-employment on the mental health of the elderly groups in different physical health states. Because for the elderly with poor physical health, their mental health is likely to be poor. If the elderly with low physical health level can improve their mental health by participating in self-employment, then self-employment can help the mental health of vulnerable elderly groups. Self-assessment of health and disease status is usually a common indicator to measure the physical health of the elderly [7, 46, 47]. The use of medical services also reflects the health status of the elderly at the level of health behavior [48]. Therefore, we selected three aspects of self-rated health, chronic disease and medical service utilization for heterogeneity analysis. From the perspective of physical health, these three aspects are interrelated. They measure the physical health of the elderly from the subjective, objective and behavioral aspects, which can more comprehensively show the difference in the impact of self-employment on the mental health of the elderly in different physical health.

Concerning self-evaluation of health, in accordance with the questionnaire, the answer “relatively healthy” or “very healthy” is defined as good, otherwise it is poor. Concerning medical service utilization, it is based on the questionnaire design, asking the interviewee, “How do you usually deal with minor illnesses?“ The answer “going to a specialist/general hospital” or “going to a village clinic/community hospital” is divided into the medical service utilization group, while the answer “buying medicines at pharmacies, using personal standing medicines for treatment, and failing to see a doctor, to wait for gradual recovery” is divided into the medical service utilization unused group. Table 6 reports the heterogeneity test results of self-employment on the mental health of the younger elderly.

The results show that the predicted coefficient of self-employment of the self-rated healthy group is significantly negative at 1% level, while that of the self-rated poor group is significantly negative at 5% level, indicating that self-employment of the younger elderly reduces their depression and promotes their mental health, regardless of their self-estimation on health. But for the elderly self-rated as healthy group, the positive impact of self-employment on mental health is greater than that of the self-rated poor group. In terms of chronic diseases, self-employment is more effective in promoting the mental health of the younger elderly with no chronic diseases. However, for the utilization of medical services, self-employment has a relatively higher impact in the low-level medical service utilization group.

**Table 6:** Heterogeneity Analysis of the Influence of Self-employed Employment on the Mental Health of the Young Elderly

Variable	Self-evaluation of health		Chronic ailment		Medical service utilization	
	Good	Discrepancy	Have	Without	Use	Unused
Self employment	-1.554***	-1.370**	-1.191***	-1.845***	-1.444***	-1.679***
	(0.308)	(0.533)	(0.0199)	(0.377)	(0.390)	(0.382)
Constant term	8.210***	9.362***	9.301***	9.334***	8.247***	9.569***
	(0.559)	(0.619)	(0.481)	(0.745)	(0.610)	(0.572)
Control variable	control	control	control	control	control	control
Virtual variables of provinces	control	control	control	control	control	control
Pseudo R2	0.248	0.129	0.178	0.266	0.205	0.219
Observed value	2025	1745	2771	999	1839	1931

Note: * * *, * * are significant at the statistical level of 1% and 5% respectively, and the standard error of robustness is in brackets

In addition to the above heterogeneous effects, whether the impact of self-employment on the mental health of different elderly people is different is worth further discussion. Based on this, this paper examines the urban-rural heterogeneity of the impact of self-employment on the mental health of the young elderly. The estimated results are shown in Table 7. It can be seen from the results in the table that the regression coefficient of self-employment in urban samples is significantly negative at the statistical level of 1%, and that in rural samples is also significantly negative at the statistical level of 1%, indicating that both the urban and rural elderly have improved their mental health level by participating in self-employment.

**Table 7:** Difference in the impact of self-employment on the mental health of urban and rural young elderly

Variable	Urban	Rural
Self employment	-1.465***	-1.463***
	(0.423)	(0.356)
Constant term	8.377***	9.307***
	(0.691)	(0.553)
Control variable	control	control
Province dummy variable	control	control
Pseudo R2	0.216	0.160
Observations	1809	1961

Note: * * *are significant at the statistical level of 1%. The brackets are robust standard errors

### Mechanism test based on KHB decomposition effect

In order to verify the intermediary mechanism of self-employment promoting the mental health of the younger elderly by increasing income and realizing self-worth, the KHB effect decomposition method was used for testing. In the definition of income, the total income of an individual in the past 12 months is used as a proxy variable to test the intermediary effect of income. In terms of self-worth variables, according to the questionnaire design, “Do you feel you have nothing to do?“ The answer “No” is assigned to 1, and “Sometimes” or “Often” is assigned to 0. Table 8 reports the estimation results based on KHB intermediary test.

The intermediary result of income effect shows that the indirect effect of income is significantly negative at the statistical level of 5%, and the predicted indirect effect accounts for about 4.21% of the total effect of self-employment on mental health, which indicates that self-employment can indirectly reduce the depression tendency of the younger elderly by increasing their economic income, thus improving their mental health. The indirect effect of self-value intermediary effect is significantly negative at the statistical level of 1%, and its indirect effect accounts for 33.49% of the total effect, which indicates that self-employment can promote the mental health of the younger elderly by realizing their self-value. Therefore, self-employment can not only directly improve the mental health of the younger elderly, but also indirectly promote their mental health by increasing their income and realizing their self-worth. Hypothesis 2 has been verified.

Further analysis shows that the contribution rate of income effect is 13.76%, and the contribution rate of self-worth is 86.24%. It can be seen that although self-employment can influence the mental health of the younger elderly through both income and self-worth, the mental health benefits brought by self-worth is greater than by improvement of economic level. It demonstrates that the spiritual satisfaction of the elderly is increasingly more important, compared to the improvement of economic level.

**Table 8:** KHB decomposition results of self-employment on mental health of young elderly people

Mediator variable	Income effect	Self-worth
Total effect	-1.451***	-0.758***
	(0.278)	(0.222)
Direct effect	-1.390***	-0.504**
	(0.278)	(0.223)
Indirect effect	-0.061**	-0.254***
	(0.025)	(0.069)
Indirect effect proportion	4.21%	33.49%
Indirect effect contribution rate	13.76%	86.24%
Control variable	control	control
Observed value	3770	3810

Note: * * *, * * and * are significant at the statistical level of 1%, 5% and 10% respectively, and the standard error of robustness is in brackets

## Discussion

How to cultivate a positive view of aging, make the elderly people feel “useful in the old age”, continue to realize their self-worth, and improve the mental health level of the elderly is a heated topic of general concern in society. This paper believes that self-employment has a significant inhibitory effect on the depression tendency of the young elderly and can promote their mental health, which is consistent with the results of Ahn [27]. Fiske et al. [49] pointed out that the decline in the level of mental health of the elderly is related to reducing social activities, and participating in valuable social activities can alleviate depressive symptoms. Self-employment provides opportunities for the elderly to remain physically and mentally active. The elderly’s choice to participate in self-employment will enable them to remain mentally, physically and socially active, and ultimately help to improve their mental health. Heterogeneity analysis shows that self-employment is beneficial to the mental health of both urban and rural elderly, and also reflects the positive effect of self-employment on the mental health of rural elderly. This result is similar to the findings of Jia et al. [15]. They found that the participation of rural elderly in local self-employment significantly improved their mental health. Therefore, encouraging self-employment is a way to cultivate the positive attitude of the elderly. At the same time, the impact of self-employment has a greater role in improving the mental health of the young elderly who have self-rated good health, no chronic disease and low level of medical service utilization. In this study, self-employment can promote the improvement of mental health of young and elderly people with better health conditions, which is consistent with the study of Sewdas et al. [8], who believe that physical health is considered an important prerequisite for working after retirement age. Therefore, the higher the level of physical health, the more able to undertake self-employment work, so as to further improve their mental health in self-employment activities. For the young elderly, the premise of self-employment is to have a high level of health [50]. The young elderly can continue to participate in labor employment only when their physical conditions permit. Therefore, the role of self-employment in promoting the mental health of the young elderly can only be better played on the premise of having certain healthy human capital [51].

From the perspective of intermediary mechanism, income promotion and self-value realization are important mechanisms for self-employment to affect the mental health of the young elderly. The contribution of income effect is relatively weak, while self-value realization plays a considerable indirect role, which shows that with the development of China’s economy, compared with economic benefits, the elderly pursue more of the reflection of the intrinsic value that self-employment brings to them. This finding was also proved in the study of Hinterlong et al. [52]. They pointed out that self-employment can alleviate depression by meeting the needs of the elderly for self-realization.

According to Labor Economics and Maslow’s Hierarchy of Needs, self-employment can meet basic living needs by increasing economic income, whereas self-employment can bring them physical and mental pleasure by satisfying spiritual needs such as self-worth, thus alleviating negative emotions such as loss, anxiety and depression caused by old age.

The policy implications of this paper are as follows: First, with the deepening of aging, self-employment of the younger elderly is generally conducive to improving their mental health level. The elderly should be encouraged and supported to actively participate in self-employment activities, continuously make contributions and improve their quality of life. The second is to refine the employment security for the elderly. The government should further formulate an active employment policy for the elderly, institute corresponding support policies for the elderly to carry out self-employment activities, provide conveniences, and reduce the burden of self-employment activities for the elderly as much as possible. The third is to steadily improve the physical health of the elderly, not only to improve the medical level, but also to spur daily physical exercise, so that the elderly can participate in self-employment in better condition. Fourth, pay attention to the satisfaction of the spiritual value needs of the elderly, and make greater efforts to publicize the tremendous contributions of self-employment that the elderly make to the society, enabling the elderly to experience the positive feedback of self-worth, thus improving the subjective initiative of the elderly to participate in self-employment, and furthering the thorough realization of the healthy aging in " feeling useful and productive for the elderly” in society.

## Conclusion

Based on the data of the 2018 China Longitudinal Aging Social Survey(CLASS), this paper examines the impact of self-employment of the younger elderly on their mental health. It is found that self-employment can significantly inhibit the depression tendency of the younger elderly and promote their mental health. The conclusion is still valid by changing the setting of the explained variables and using Propensity Score Matching method to check the counterfactual. Heterogeneity analysis shows that the influence of self-employment has a greater effect on ameliorating the mental health of the younger elderly who are self-rated as healthy, have no chronic diseases and have low-level medical service utilization. From the perspective of intermediary mechanism, income growth and self-worth realization are the important mechanisms that self-employment affects the mental health of the younger elderly, among which the contribution of income effect is relatively weak, while self-worth realization performs considerable indirect influence. It demonstrates that with the development of China’s economy, compared with economic gains, the elderly pursue more intrinsic value that self-employment brings.

Of course, this research also has some limitations that can be solved in future research. The details are as follows: First, we selected 2018 CLASS data as the research sample of this paper. However, it is now 2023, and the COVID-19 epidemic has had a great impact on the labor market. The epidemic may have some moderating effect on the self-employed employment of the elderly and their mental health. At the same time, the impact of self-employed employment on the mental health of the elderly is a dynamic process. Therefore, future research needs to use updated and more data to further expand and verify the relationship between the two in more detail. Second, there are many types of self-employment, such as opportunity self-employment and survival self-employment. Different types of self-employment may also have different effects on the mental health of the elderly. Unfortunately, the 2018 CLASS database did not distinguish the types of self-employment. Based on the current data, we cannot conduct in-depth research on this, which is the limitation of this paper. This will also become the direction of further research in the future, so as to better enrich the research in this field. Third, the sample size can be further expanded. From the perspective of objective reality, the sample of the elderly self-employed group that can be collected from the existing data is low, resulting in a low proportion of self-employed samples in the total sample, which is one of the shortcomings of this study. However, we believe that with the implementation of China’s delayed retirement policy, the number of self-employed elderly groups will increase in the future data. In the future, we will continue to track the latest data and research trends to continue to carry out in-depth research.

## Data Availability

The data is not publicly available. The datasets used and/or analysed during the current study available from the corresponding author on reasonable request.
